# Response to Perrier and Charmantier: On the importance of time scales when studying adaptive evolution

**DOI:** 10.1002/evl3.112

**Published:** 2019-04-04

**Authors:** Mirte Bosse, Lewis G. Spurgin, Veronika N. Laine, Ella F. Cole, Josh A. Firth, Phillip Gienapp, Andrew G. Gosler, Keith McMahon, Jocelyn Poissant, Irene Verhagen, Martien A. M. Groenen, Kees van Oers, Ben C. Sheldon, Marcel E. Visser, Jon Slate

**Affiliations:** ^1^ Department of Animal Ecology Netherlands Institute of Ecology (NIOO‐KNAW) Wageningen The Netherlands; ^2^ Wageningen University and Research—Animal Breeding and Genomics Wageningen The Netherlands; ^3^ School of Biological Sciences University of East Anglia United Kingdom; ^4^ Edward Grey Institute, Department of Zoology University of Oxford United Kingdom; ^5^ Department of Ecosystem and Public Health, Faculty of Veterinary Medicine University of Calgary Canada; ^6^ Department of Animal and Plant Sciences University of Sheffield United Kingdom

**Keywords:** Adaptation, genetic architecture, genomics, natural selection

Inferring adaptation and evolutionary change by combining data from field studies and genomics is an exciting new area in evolutionary biology but also presents challenges. These challenges are particularly acute when the focal trait has a polygenic architecture, because many long‐term field studies are sample‐size‐limited compared to studies of humans and model organisms, making the detection of loci that contribute to trait variation difficult. In a recent comment, Perrier and Charmantier (2018); hereafter P&C, highlight these issues and draw attention to several analyses described in our recent publication (Bosse et al. 2017) on the evolution of longer bill length in UK populations of the great tit *(Parus major*). While we support the overall message of P&C – that caution should be exercised when making inferences about long‐term evolutionary trends from shorter ecological time series – we also address some of the specific criticisms that P&C raised about the analyses described in Bosse et al. (2017).

P&C's comments can broadly be split into two sets of queries. The first considers how phenotypic variation is distributed in space and time. The second explores how signatures of selective sweeps can be sensitive to local (in the genomic sense) variation in recombination rate.

## Spatio‐Temporal Patterns

### TIME SERIES OF BILL LENGTH

In Bosse et al. (2017) we presented a number of analyses supporting the contention that bill length in UK populations had been under positive selection (see below), one of which was the observation that, over a 25‐year time series, bill length had significantly increased. We performed a linear regression of bill length on year of birth–‐in hindsight a mixed model with year fitted both as a fixed effect (to detect any temporal trend) and as a random effect (to account for annual differences in bill length variation) would have been a better choice of model. P&C have investigated the relationship in more detail. After inspection of Fig. 4B of Bosse et al. (2017), P&C observed a downturn in the mean bill length‐year relationship, which they subsequently analyzed more formally by a breakpoint analysis, aimed at identifying whether there was a rapid change in the mean bill length temporal trend. P&C showed that the significant increase in the bill length linear model was dependent on the inclusion of the first five years of the dataset (1982–1986) in the analysis; if these years are excluded from the model, bill length has significantly decreased. P&C then argued that the data cannot be used to support evidence of contemporary (1982–2007) evolution of bill length, and especially that the data cannot be used to support the idea that bird feeder use was driving a contemporary increase in bill length. They also suggested that further research should investigate whether an apparent decline in bill length, starting in 1986, could be caused by a genetic change, phenotypic plasticity, or a change in the measuring process.

**Figure 1:**
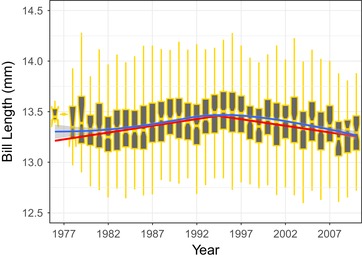
Temporal pattern of bill length in Wytham Woods, using the dataset described in Table S3 of Bosse et al. (2017). Loess curve (blue line) is fitted using the ggplot2 stat_smooth() function. Slope estimates from *segmented* breakpoint analysis are shown as a red line. Note that bill length appeared to be increasing from the start of the time series until ∼1995 and has decreased from that point. Bill length measurements are corrected for sex, age of bird, month measured, and whether the bird was a resident or immigrant to Wytham Woods. All birds were measured by AGG.

We do not dispute that there is an apparent decline in bill length during the latter part of the time series–that pattern holds even when a mixed model with year included as a random effect is fitted (see Supplementary Information). However, we suggest that the trend should be interpreted cautiously. The authors cite the work of Rosemary and Peter Grant as their inspiration for searching for sudden changes in the trajectory of bill morphology in the great tit time series, but that work was motivated by selection witnessed after a sudden climatic event; an *El Nino* event in 1982–1983 causing exceptionally heavy rainfall (Grant and Grant 1993). In the analysis in Bosse et al. 2017 however, there is no a priori reason to expect bill length to have started to change after 1986. Instead, P&C's analysis was motivated by a posthoc inspection of the figure in Bosse et al. 2017. Moreover, the conclusion that bill length declined after 1986 is sensitive to the dataset used. In the supplementary material of Bosse et al. (2017) we described a longer (1976–2010) and larger (9980 records, from 5145 birds) dataset, comprising birds measured throughout the year; the series described in the main text of Bosse et al. (2017) and reanalyzed by P&C was a subset of 2489 birds that were measured in May or June. Using a linear‐mixed model framework, the larger dataset also shows an increase in mean bill length (Table S3 in Bosse et al. 2017). Adopting a similar approach used by P&C, we searched the larger dataset, using the R package segmented (Muggeo 2008), for a breakpoint where bill length switched from an increasing trend to a declining one. The larger dataset also appears to be consistent with bill length increasing over the first half of the time series, followed by a decline in the second half (Fig. 1; null hypothesis of no change in slope has *P* < 0.0001). However, the decline in bill length starts between 1995 and 1996. Therefore, the period where bill length was increasing spans an 18‐year period rather than a five year one, and the decline may have started in 1995 rather than 1986. Great tit bill size, especially length, shows strong fluctuations between years and seasons as a response to diet (Gosler 1987). Thus, we think it is unwise to place too much emphasis on the possibility of an evolutionary or plastic response to an unknown environmental change occurring in 1986 (or any other year).

There is additional evidence that evolution of longer bills has been occurring over a longer, yet nonetheless recent, period in the UK population. In Fig. 4A of Bosse et al. 2017, we used a sample of 291 museum specimens of great tits to describe a difference in bill length between UK and mainland European populations. P&C show that there is no temporal variation in bill length in the museum samples collected in the UK between 1850 and 2007. However, given the large within‐year variation in bill‐length (Gosler 1987), the power to detect a trend is very low in this sample (*n* = 177) in comparison to the contemporary data, which is why we declined to test for it in Bosse et al. (2017). Ideally, we would compare genomic breeding values from museum and contemporary birds to evaluate whether there was an underlying genetic change in bill length, but genomic data are not currently available from museum specimens.

In Bosse et al. (2017) we were careful not to make the argument that the recent Wytham time series implicated the role of bird feeders in the evolution of longer bills. Rather, the increase in bill length was one piece of evidence for the relatively recent evolution of longer bills, along with: (i) signatures of selection being more prevalent at genes associated with craniofacial morphology and palate development; (ii) loci affecting bill length being found in selective sweep regions more often than expected by chance; (iii) UK populations exhibiting longer bills than other European populations; (iv) alleles causing longer bills being associated with greater fitness; and (v) the use of haplotype‐based tests of selective sweeps that are sensitive to relatively recent selection.

### SPATIAL PATTERNS

P&C have explored the spatial museum data presented by Bosse et al. (2017) in more detail. In Bosse et al. (2017) the comparison was between UK and mainland European birds, with UK birds having bills approximately 0.4 mm longer than European birds. P&C compare the UK birds to each of the mainland European countries (*n* = 11 countries). UK birds had longer bills than birds from these other countries, but were only significantly longer than birds from three countries (the Netherlands, France, and Italy). However, the sample sizes for the mainland countries ranged from two to 33, so the power to detect differences from the UK population was very low. P&C argue that because the bill length across different countries is not bimodal (i.e., with UK birds all in one distribution and birds from all other populations in a second distribution), that is evidence against recent evolution of bill length in the United Kingdom. This argument is incorrect. A highly polygenic trait is likely to exhibit a continuous distribution between populations, even if one (or more) population is evolving by natural selection toward a larger mean, simply due to the effects of genetic drift and environmental variation being unequal in all populations.

**Figure 2:**
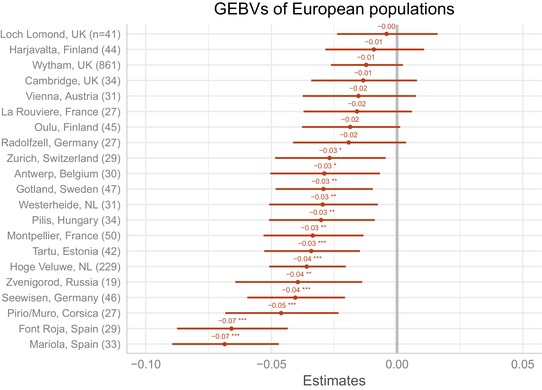
Genomic estimated breeding values (and SEs of bill length in European great tit populations. GEBVs were estimated from the loci identified by Bosse et al. (2017) as being under selection. The training population was a set of 89 phenotyped birds from Wytham Woods (UK). Test populations include unphenotyped Wytham birds. Sample sizes are included in parentheses. Most mainland European birds have lower GEBVs than Wytham Woods and other UK populations. There is some evidence that Finnish populations also have GEBVs for longer bills. Genomic data from populations outside of the United Kingdom and the Netherlands are described elsewhere (Spurgin et al. 2019).

Ideally, the effects of the environment on spatial variation in bill length would be removed or reduced by comparing the genetic component of bill length between populations. In Fig. S7 of Bosse et al. (2017) we compared the breeding values of the United Kingdom and the Netherlands populations. As part of an ongoing effort to characterize genetic variation across the species’ distribution (the Great Tit HapMap Project; (Spurgin et al., 2019) we have genotyped birds from >20 European populations with the same SNP chip (Kim et al. 2018) as the one used in Bosse et al. (2017). Based on those data, here we ask whether the loci identified as being under selection in the United Kingdom could cause UK populations to have longer bills than mainland European populations. By using genotyped and phenotyped birds from Wytham as a training population, we performed genomic prediction of bill length in the other European populations. The results (Fig. 2) show that loci under selection cause UK populations to have greater genomic estimated breeding values (GEBVs), that is genetically longer bills, than most other European populations. Note that UK populations additional to the one at Wytham Woods are included in this dataset. It is also striking that a Finnish population has larger GEBVs than other European populations, suggesting that this population may also be experiencing selection for longer bills. All other mainland European populations have lower mean GEBVs (often significantly lower) than UK populations. Notably, the museum birds from Finland had longer bills than the other European mainland populations (see Fig. 4 of P&C), although the sample size is very small (*n* = 4). We interpret these results cautiously, because the accuracy of genomic prediction can depend on population structure, LD pattern (between markers and unknown causal loci), trait architecture, and genotype by environment interactions. In particular, a comparison of samples that are temporally separated by many generations may require recalibration of the relationship between SNP genotypes and phenotypic variation, as the marker‐causal loci LD relationships may be altered by recombination (Habier et al. 2009). However, cross‐population polygenic scores tend to be most reliable when population differentiation is low, as is the case here (Bulik‐Sullivan et al. 2015; Berg et al. 2018).

## Genomic Evidence of Selection at COL4A5

We now switch our focus to the genomic arguments made by P&C. In Bosse et al. (2017), much of the focus on genomic signatures of selection was on the *COL4A5* locus. This region showed evidence of a selective sweep in the UK population, and was also one of the region's most strongly associated with bill length variation in the United Kingdom, with the selected haplotype associated with longer bills. Further, the positively selected haplotype was associated with an increased production of fledglings and an increased number of visits to feeders. It is important to note, though, that the observation that selected regions explained more variation in bill length than expected by chance was not dependent on the *COL4A5* region. Neither was the observation that the Gene Ontology (GO) term most significantly overrepresented among the selected loci was palate development. Thus, the overall conclusion that bill length has been a target of selection is not critically dependent on *COL4A5* being a contributor to the polygenic architecture of bill length variation.

P&C convincingly show that *COL4A5* is in a region of low recombination. They point out, highlighting recent evidence (Burri 2017; Comeron 2017), that background selection in regions of low recombination can give spurious evidence of selective sweeps in F_ST_ outlier based tests (the eigenGWAS test used by Bosse et al. 2017 falls into this category). P&C use simulations to show that eigenGWAS tests could easily give a signal that looks like positive selection, when instead linked background selection is acting within populations. They argue that the apparent adaptive evolution at *COL4A5* could be an artefact of background selection in a region with limited recombination; the argument is given further credibility by the observation that the same region shows a similar signal in collared flycatchers, where recombination is also very limited (Burri et al. 2015). P&C raise an important and valid point; however, for reasons we outline below, we remain confident that it has been one of the loci involved in adaptive evolution of longer bills in UK populations.

**Figure 3:**
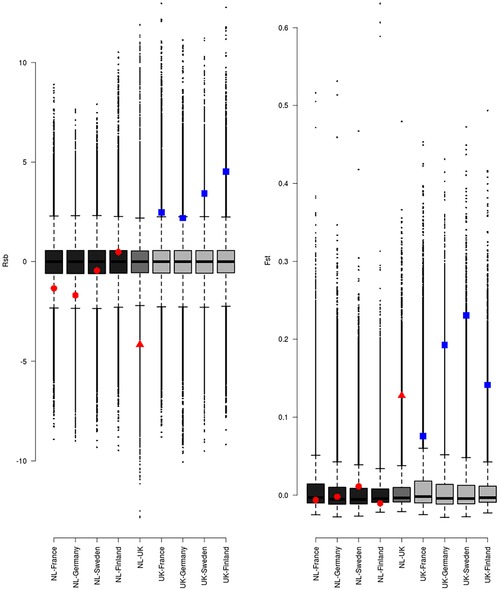
Rsb (A) and F_ST_ (B) statistics for the *COL4A5* locus, relative to the genomewide distribution of each statistic. Comparisons are between pairs of populations; either Veluwe and Wytham, or one of those two populations and another population from Europe (one of Montpellier, France; Seewisen, Germany; Gotland, Sweden; Harjavalta, Finland). The comparison between the Veluwe and Wytham populations, reported in Bosse et al. (2017), are indicated by red triangles. Comparisons between one of Veluwe/Wytham and another European population, are indicated by red circles/blue squares, respectively. For both tests, *COL4A5* is an outlier in UK‐European comparisons, and is not an outlier in comparisons between two European populations. Note that in (A) a negative value of Rsb in the NL‐UK comparison, and a positive value in all of the other UK comparisons is consistent with the haplotype associated with long bills being under positive selection in the UK population.

The evidence for a selective sweep at *COL4A5* came not only from eigenGWAS and F_ST_ outlier locus analyses, which are sensitive to local recombination rates, but also from an Rsb test (Tang et al. 2007), that compares haplotype homozygosity between two populations (see Fig. S8 of Bosse et al. 2017). Rsb and similar test statistics such as the cross‐population extended haplotype homozygosity (XP‐EHH) are considered robust to local recombination rate, provided the recombination landscapes are similar in the two populations being compared (Tang et al. 2007; Enard et al. 2014). We have previously shown through linkage maps independently constructed in the United Kingdom and the Netherlands populations that recombination landscapes are highly conserved between great tit populations (van Oers et al. 2014). Furthermore, if P&C were correct and the evidence of selection at *COL4A5* in the United Kingdom was an artefact caused by background selection in a region of low recombination, then there is a clear prediction: F_ST_ or EigenGWAS tests as well as haplotype homozygosity tests (Rsb) would show strong signatures of selection between the Netherlands population and other (non‐UK) populations. We find no evidence for such a pattern in either Rsb or Fst (Fig. 3). Thus, the data are more consistent with positive selection acting at *COL4A5* in the United Kingdom rather than spurious signatures of a sweep caused by background selection in a low recombining region operating in all great tit populations. Finally, there is no reason to think that the association between SNPs in and around *COL4A5* and bill length is an artefact caused by low local recombination rates. Although regions with low recombination rates (and therefore high linkage disequilibrium) will have enhanced power to detect genuine causal variants in a GWAS (Visscher et al. 2017), there is no reason why they should be more prone to false positive associations. We note that the region has not been associated with other traits studied in the Wytham population in previous GWAS studies (Santure et al. 2013; Santure et al. 2015; Kim et al. 2018).

In summary, we are in broad agreement with the general points made by P&C. Pinning down the time scale of adaptive evolution remains a challenging problem, especially when there have been numerous genomic regions driving an evolutionary response to selection. We hope to make progress with understanding the evolution of great tit bill length by genomic analysis of museum samples and other European populations. We remain cautious about supplementary feeders being the cause of longer bill length evolution and confirming (or ruling out) that explanation will require careful experimentation and ecological study, alongside accurate dating of when longer bill length evolution started. Despite the caveats identified by P&C, the evidence that great tit bill length has evolved under recent positive selection in UK populations clearly remains, as does the evidence that *COL4A5* is likely to be one of many loci involved in this adaptation. One clear message arising from P&C and this paper is that attempts to make inference about polygenic adaptation from any single line of evidence are likely to be inconclusive. Instead, careful formulation and testing of hypotheses that incorporate different types of data are more likely to prove incisive. More generally, combining multiple interdisciplinary approaches is the key to understanding the mechanisms involved in local adaptation.

Associate Editor: Z. Gompert

## Supporting information


**Table S1**. Mixed model analysis of data presented in Fig 1 of P & C and Fig 4b of Bosse et al. 2017.

## References

[evl3112-bib-0001] Berg, J. J. , A. Harpak , N. Sinnott‐Armstrong , A. M. Joergensen , H. Mostafavi , Y. Field , et al. 2018 Reduced signal for polygenic adaptation of height in UK Biobank. BioRXiv. 10.1101/354951 PMC6428572 30895923

[evl3112-bib-0002] Bosse, M. , L. G. Spurgin , V. N. Laine , E. F. Cole , J. A. Firth , P. Gienapp , et al. 2017 Recent natural selection causes adaptive evolution of an avian polygenic trait. Science 358:365–368. 29051380 10.1126/science.aal3298

[evl3112-bib-0003] Bulik‐Sullivan, B. , P. Loh , H. K. Finucane , S. Ripke , J. Yang , Schizophrenia Working Group of the Psychiatric Genomics Consortium, N. Patterson, M. J. Daly, M. A. L. Price and B. M. Neale . 2015 LD score regression distinguishes confounding from polygenicity in genomewide association studies. Nat. Genet. 47:291–295. 25642630 10.1038/ng.3211 PMC4495769

[evl3112-bib-0004] Burri, R. 2017 Interpreting differentiation landscapes in the light of long‐term linked selection. Evol. Lett. 1:118–131.

[evl3112-bib-0005] Burri, R. , A. Nater , T. Kawakami , C. F. Mugal , P. I. Olason , L. Smeds , et al. 2015 Linked selection and recombination rate variation drive the evolution of the genomic landscape of differentiation across the speciation continuum of *Ficedula flycatchers* . Genome Res. 25:1656–1665. 26355005 10.1101/gr.196485.115 PMC4617962

[evl3112-bib-0006] Comeron, J. M. 2017 Background selection as null hypothesis in population genomics: insights and challenges from Drosophila studies. Philos. Trans. R Soc. B Biol. Sci. 372:20160471. 10.1098/rstb.2016.0471 PMC5698629 29109230

[evl3112-bib-0007] Enard, D. , P. W. Messer , and D. A. Petrov . 2014 Genome‐wide signals of positive selection in human evolution. Genome Res. 24:885–895. 24619126 10.1101/gr.164822.113 PMC4032853

[evl3112-bib-0008] Gosler, A. G. 1987 Pattern and process in the bill morphology of the Great tit Parus‐major. Ibis 129:451–476.

[evl3112-bib-0009] Grant, B. R. , and P. R. Grant . 1993 Evolution of Darwin finches caused by a rare climatic event. Proc. R Soc. B Biol. Sci. 251:111–117.

[evl3112-bib-0010] Habier, D. , R. L. Fernando , and J. C. M. Dekkers . 2009 Genomic selection using low‐density marker panels. Genetics 182:343–353. 19299339 10.1534/genetics.108.100289 PMC2674831

[evl3112-bib-0011] Kim, J. M. , A. W. Santure , H. J. Barton , J. L. Quinn , E. F. Cole , C. Great Tit HapMap , et al. 2018 A high‐density SNP chip for genotyping Great tit (*Parus major*) populations and its application to studying the genetic architecture of exploration behaviour. Mol. Ecol. Resour. 18:877–891. 29573186 10.1111/1755-0998.12778

[evl3112-bib-0012] Muggeo, V. M. R. 2008 Segmented: an R package to fit regression models with broken‐line relationships. R News 8:20–25.

[evl3112-bib-0013] Perrier, C. , and A. Charmantier . 2018 On the importance of time scales when studying adaptive evolution. Evol. Lett. 10.1002/evl3.86 PMC6546376 31171979

[evl3112-bib-0014] Santure, A. W. , I. De Cauwer , M. R. Robinson , J. Poissant , B. C. Sheldon , and J. Slate . 2013 Genomic dissection of variation in clutch size and egg mass in a wild great tit (*Parus major*) population. Mol. Ecol. 22:3949–3962. 23889544 10.1111/mec.12376

[evl3112-bib-0015] Santure, A. W. , J. Poissant , I. De Cauwer , K. van Oers , M. R. Robinson , J. L. Quinn , et al. 2015 Replicated analysis of the genetic architecture of quantitative traits in two wild great tit populations. Mol. Ecol. 24:6148–6162. 26661500 10.1111/mec.13452 PMC4738425

[evl3112-bib-0016] Spurgin, L. G. , M. Bosse , F. Adriaensen , T. Albayrak , C. Barboutis , E. J. Belda , et al. 2019 The great tit HapMap project: a continental‐scale analysis of genomic variation in a songbird. bioRXiv 10.1101/561399 38747336

[evl3112-bib-0017] Tang, K. , K. R. Thornton , and M. Stoneking . 2007 A new approach for using genome scans to detect recent positive selection in the human genome. PLoS Biol. 5:1587–1602. 10.1371/journal.pbio.0050171 PMC1892573 17579516

[evl3112-bib-0018] van Oers, K. , A. W. Santure , I. De Cauwer , N. E. M. van Bers , R. P. M. A. Crooijmans , B. C. Sheldon , et al. 2014 Replicated high‐density genetic maps of two great tit populations reveal fine‐scale genomic departures from sex‐equal recombination rates. Heredity 112:307–316. 24149651 10.1038/hdy.2013.107 PMC3931172

[evl3112-bib-0019] Visscher, P. M. , N. R. Wray , Q. Zhang , P. Sklar , M. I. McCarthy , M. A. Brown , and J. Yang . 2017 10 years of GWAS discovery: biology, function, and translation. Am. J. Hum. Genet. 101:5–22. 28686856 10.1016/j.ajhg.2017.06.005 PMC5501872

