# Supplementary material for: Response to Perrier and Charmantier: On the importance of time scales when studying adaptive evolution
Source: Evol Lett. 2019 Apr 4;3(3):248–53. doi: 10.1002/evl3.112 (PMC6546378; doi:10.1002/evl3.112)
Supplement: Supplementary file 1 — Table S1. Mixed model analysis of data presented in Fig 1 of P & C and Fig 4b of Bosse et al. 2017. [file EVL3-3-248-s001.docx]

Response to Perrier and Charmantier: On the importance of time scales when studying adaptive evolution

**Supplementary material**

In both Bosse et al. (2017) and Perrier and Charmantier (2018), the analyses of temporal trends in great tit bill length at Wytham Woods used a linear regression approach. However, these tests can be anti-conservative if the within year variation in bill length is variable between years. A more robust approach would be to fit a mixed effects model with birth year included as a random effect in the model, as well as a fixed effect. We fitted this model, with birth year included as a second order polynomial, as in Perrier and Charmantier (2018). The model was run in the R package MCMCglmm (Hadfield 2010), using the default number of iterations (13,000), burnin period (3000) and thinning interval (every 10^th^ iteration). Note that the birth year polynomial term has strong statistical support, consistent with bill length increasing but then declining. i.e. the general pattern described by Perrier and Charmantier.

Table S1: Mixed model analysis of data presented in Fig 1 of P & C and Fig 4b of Bosse et al. 2017. Data are available on Dryad at https://doi.org/10.5061/dryad.p03j0

| Random Effects | Variance |  |
| --- | --- | --- |
| Birth year | 0.005 (0.001-0.010) |  |
| Residual | 0.190 (0.179-0.199) |  |
| Fixed Effects | Estimate (95% credible interval) | pMCMC |
| Intercept | 13.68 (13.64-13.72) | < 0.001 |
| Sex | -0.22 (-0.25 - -0.19) | <0.001 |
| Birth Year | 1.26 (-0.14 -3.12) | 0.102 |
| (Birth Year)^2^ | -2.62 (-4.10 - -0.86) | 0.004 |

Hadfield, J. D. 2010. MCMC Methods for Multi-Response Generalized Linear Mixed Models: The MCMCglmm R Package. Journal of Statistical Software **33**:1-22.
